# Addressing consequences of school closure on oral health care of children during COVID-19

**DOI:** 10.3389/fped.2022.725977

**Published:** 2022-07-22

**Authors:** Radhika Chhibber, Richa Shrivastava, Madhura Tandale

**Affiliations:** ^1^Faculty of Dental Medicine and Oral Health Sciences, McGill University, Montreal, QC, Canada; ^2^Faculty of Dentistry, Université de Montréal, Montreal, QC, Canada

**Keywords:** oral health promotion interventions, child nutrition, COVID-19, hunger alleviation programs, early childhood caries

## Introduction

Children are less likely to be infected by COVID-19 and are more likely to be asymptomatic, yet they have faced the biggest psychological and behavioral impacts resulting from the pandemic (1). It has affected many children across countries, including caste, gender, race, and religion. The pandemic has placed children from low socioeconomic and vulnerable families in high-income groups at risk (2). A high proportion of these populations live in residences which are exposed to overcrowding, making social distancing virtually impossible (3). The school closures and the decision to shift traditional classrooms to online models have increased learning inequalities among children and pushed many children out of school permanently due to the digital division (4). Aside from learning, the absenteeism of schooling holds long-term consequences on children's nutrition and overall health, including oral health (3).

A systematic review has reported that school-based oral health programs at the regional and national levels significantly improve the oral hygiene of the children in equitable manner (5). However, the suspension of oral health programs due to the pandemic has had a substantial influence on children's access to dental care, particularly among the most vulnerable. An exploratory study by Meyer and Danish on assessing impact of COVID-19 on preventive dental service utilization by the USA children reported significant decrease in service utilization rate at the dental visits and well-child visits (6). Furthermore, a survey on measuring the COVID-19 impact on the planning and delivery of state-funded school oral health programs in the United States found that pausing and delaying such programmes during the pandemic can lead to an increase in children's oral diseases in foreseeable future, leading to even more inequalities (3).

Budgeting is critical in the current situation even beyond the epidemic to ensure a comprehensive oral health education programme for school children. The pandemic has provided governments with valuable policy lessons for dealing with such situations, as well as the opportunity to revamp the system to be better prepared to deal with them. The government should put forward innovative platforms that are easy-to-access and economical school-based educational interventions that can improve oral health among school children, especially in countries with a developing oral health care system. However, the overall direction of allocations should not be limited to addressing pandemic-related issues but should go beyond that. This article highlights some of the issues concerning child nutrition and oral health care as a result of school closures in India that require immediate attention.

### School-based child nutrition programs in India

Schools are an ideal platform for health promotion activities because they are accessed by most of the child population and therefore offer the largest target group (3). Moreover, schools provide a supportive environment at a relatively early stage of a child's life and continuously screen and support healthy behaviors over time. Many nations have school-based health and nutrition programs in place that incorporate school meals, deworming, vitamin and mineral supplementation, and so on (7). This type of intervention offers potential avenues to foster good dietary habits and lifestyle choices, as well as involve parents and the community in childhood malnutrition prevention (8).

In India, assistance programs that help to reduce food insecurity in educational settings are the Take Home Ration (9), Supplementary Nutrition Programme, or Mid-Day Meal (10). The Mid Day Meal is an important part of Indian children's diet. It is one of India's most concerted efforts, with massive advantages such as avoiding classroom hunger, improving school attendance, and tackling malnutrition (10). This programme, however, has far broader implications because it is one of the most important ways to ensure children's nutritional security.

### Food insecurity during COVID-19 crisis

In India, malnutrition levels have remained persistently high. According to the *State of Food Security and Nutrition in the World* 2020 report, “28% of the total malnourished population live in India that accounts for about 30% of the world's stunted children and nearly 50% of the severely wasted children under the age of five” (11, 12). As per the *Comprehensive National Nutrition Survey* 2016-2018, the prevalence of stunting among out-of-school children is higher than those going to school, with one-quarter of adolescents having low Body Mass Index scores, which ultimately link to being underweight (13).

Prior to the COVID_19 outbreak, India had the largest population of people suffering from food insecurity, with around 22% of global food insecurity coming from India (1). Further, food insecurity in India increased by 3.8% points between 2014 and 2019 (14).The COVID-19 pandemic has further aggravated malnutrition among the most susceptible households due to disrupted essential nutrition services, reduced income, restricted accessibility, and affordability of nutritive food, and reduced physical activity (11, 13). Furthermore, it had an impact on children's health services and immunization services. The disruption of health services caused by COVID-19 is estimated to increase child malnutrition by 10–20%, with an additional 6,000 children dying daily from this preventable cause (10).

Usually, school feeding programs come in two forms: school meals and THRs. However, the ongoing pandemic crisis has raised barriers to the accessibility of nutrition schemes. The children enrolled under such feeding programs have suffered as a result of the closure of schools across the country due to lockdown. The Mid Day Meal scheme faced massive challenges when schools and Anganwadis were shut down. According to the *Save the Children survey*, “one in every three households did not receive their take-home ration (THR) in India during the pandemic; close to two-fifths of the household reported that their children are not receiving mid-day-meal and are therefore becoming weak“ (15). As a result, the large population of children who rely on school meals for the majority of their nutritional needs are at risk of physical and cognitive harm as a result of service disruption and school dropout.

Several approaches were taken to school-based child nutrition programs during the COVID-19 outbreak. For example, where previous feeding methods may have involved many children in a large room, amendments were made to provide socially distanced feeding, including spreading children across different rooms (16). UNICEF reported that 39% of the 110 responding countries had introduced alternative approaches to school-feeding programs during the pandemic, such as cash transfers and multimodal approaches (home delivery, THRs and cash transfers) (17).

The Government of India is attempting to address this situation by enacting various policies, social security measures, budgetary allocations, for example, the COVID-19 packages for the health of Rs. 15,000 crores in April 2020, public distribution system relief package, which includes free food grains for people covered under the National Food Security Act (10). State governments are undertaking all efforts to ensuring the distribution of take-home ration door to door with the support of community workers such as ASHAs and Anganwadi workers (18). Although these initiatives are implemented to give some relief to some communities in the society, yet there seems to gap within them that needs to be addressed.

Children identified as having more complex healthcare needs (CSHCN) often have several chronic physical, developmental, behavioral or emotional conditions, as well as functional limitations (19). More than 43.5% of CSHCN had conditions that required additional services (i.e., medical, mental health, speech therapy) alone or along with the need for prescription medication, while over a quarter of CSHCN (25.6%) had a functional limitation (20). These challenges may have an impact on the ability of families to remain food sufficient, yet few studies have examined this question.

This is an important omission because research has shown that financial hardships often occur among families raising CSHCN (21). This may be due to factors such as increased out-of-pocket expenses for special foods, equipment, or medications (22–25).

While households with economic status lack access to fresh affordable food, and suffer from higher incidence of food insecurity, might cause high diets in carbohydrates which leads to increased risk factors for dental caries (26–28). Studies show that food insecure households have a higher incidence of dental caries, in comparison to food secure households (22, 28). Various studies have been conducted to assess the effect of food insecurity on oral health of children in various countries; however, the literature on this is limited as far as India is concerned. Scarce studies on the burden of the problem poses a hurdle in formulating strategies to combating this epochal issue.

Studies have also shown that adults in families with food insecure households are more susceptible to heart disease, diabetes, hypertension, increased inflammation and obesity (29–31). Moreover, children in families experiencing food insecure are more prone to psychosocial, cognitive and behavioral problems as well as a reduction in the intake of important nutrients and developmental deficiencies (32–35). Deprivation of certain nutrients and Inadequate intake of food are also proven to affect oral health (36). Severe enamel hypoplasia and chronic periodontal disease are some evident oral health problems associated with a lack of vitamin D, scorbutic gingivitis (scurvy) is associated with vitamin C deficiency and as dental caries is associated with a carbohydrate-rich diet (37, 38). Furthermore, nutrients deficient diet and high in carbohydrates, individuals in situations of food insecure may experience poor oral health. Studies have shown that unmet oral health care need was higher in children with more severe disabilities and those from lower income families (21). Many children with special healthcare needs are predisposed to poor oral health owing to sugary medications, preference for soft foods, inadequate physical activity, more screen time and cognitive and physical impairments that are barriers to hygiene (31, 32). Along with an inadequate access to preventive oral health care.

### School preventive oral health programs in COVID-19

Oral health represents a significant role in sustaining the general wellness and wellbeing of an individual. The oral health of the child depends upon nutrition in many ways. For example, proper intake of nutrients through diet influences orofacial-related development, periodontal health, and oral infections or diseases. The child's nutrition is critical in maintaining the integrity of oral health as well as preventing the progression of oral diseases (39). Despite advancements, oral diseases continue to cause discomfort, ache, sleepless nights and loss of school days (40). Moreover, the COVID-19 outbreak has significantly impacted oral health care services, with 60% of countries reporting partial disruption and 17% reporting severe/complete disruption (41). As a result, oral health disparities are widening around the world.

The pandemic has provided an opportunity to shine a spotlight more on oral health promotion strategies and approaches which help to reduce the risk of oral health diseases and to develop personal skills to maintain healthy lifestyles. World Health Organization has also recommended preventive dental services via school oral health programs which includes screening, fluoride application, sealants and oral hygiene instructions (5). These preventive programs are imperative in preventing and controlling tooth decay in school children and improving children's oral health. Oral health education has been deemed effective in enhancing knowledge, attitudes and practices of oral health in order to target the reduction of plaque, bleeding and other oral diseases (42). According to this research, oral health education is to be provided by professionals, with settings ranging from school, home, health centers, clubs and nursing homes, and techniques including videos, demonstrations and written content, or a mixture of these methods (42).

School closures as a result of COVID-19 have had significant effects on oral health in children. Research on this matter in the US has found that children in 2020 were 75% more likely to have poor oral health than they were in 2019, with dental visits down 27%, reducing opportunities for prevention (43). These findings were consistent across socioeconomic groups and different demographics, and the same differences were not found between 2019 and 2018. Currently, the literature on the effect of COVID-19-related school closures on oral health is sparse, so attention must be drawn to this area in order to understand the impacts on both oral health and nutrition in school children.

## Recommendations

It is proposed that attention is drawn to the consideration of oral health in school children to become a priority, along with nutrition, during and beyond the pandemic. Teachers and educators should continue to assist parents and caregivers and provide supervision on healthy diets and oral hygiene. Research should seek to identify potential risk factors associated with food insecurity, effects of school closure and new barriers to oral health in children, whilst also collecting and reporting on data on intervention strategies for child nutrition and oral health during the pandemic. The government should increase funding for such research activities and implement the relevant policies and guidelines on the back of scientific evidence. This research will enable policy makers to engage teachers, stakeholders, dental professionals and more to implement scientifically refuted approaches to child nutrition and oral health.

## Conclusion

School closure and transitions to hybrid learning have led to a suspension of school-based programs, which in turn interrupts children's access to the nutritious food source they once relied on during the school week, which is a driving force to increased food insecurity. Hence, policy focus should be on nutrition safety and oral health in children in order to support post-pandemic recovery. It is crucial to examine whether nutritional requirements and oral health needs of the children are being met both during the pandemic and after.

## Author contributions

RC: conceptualization and writing. MT and RS: editing. All authors listed have made a substantial, direct, and intellectual contribution to the work and approved it for publication.

## Conflict of interest

The authors declare that the research was conducted in the absence of any commercial or financial relationships that could be construed as a potential conflict of interest.

## Publisher's note

All claims expressed in this article are solely those of the authors and do not necessarily represent those of their affiliated organizations, or those of the publisher, the editors and the reviewers. Any product that may be evaluated in this article, or claim that may be made by its manufacturer, is not guaranteed or endorsed by the publisher.
